# Molecular Mechanism for Selective Cytotoxicity towards Cancer Cells of Diselenide-Containing Paclitaxel Nanoparticles

**DOI:** 10.7150/ijbs.34878

**Published:** 2019-07-03

**Authors:** Jing Li, Yue Gu, Wei Zhang, Cui-Yu Bao, Cai-Rong Li, Jing-Yi Zhang, Tao Liu, Shuai Li, Jia-Xi Huang, Zhi-Gang Xie, Shu-Cheng Hua, Ying Wan

**Affiliations:** 1 Hubei Province Key Laboratory on Cardiovascular, Cerebrovascular, and Metabolic Disorders, Hubei University of Science and Technology, Xianning, Hubei 437100, P. R. China,; 2 Department of Reparatory and Critical Care Medicine, the First Affiliated Hospital of Jilin University, Changchun 130021, P. R. China,; 3 State Key Laboratory of Polymer Physics and Chemistry, Changchun Institute of Applied Chemistry, Chinese Academy of Sciences5625 Renmin Street, Changchun, Jilin 130022, P. R. China; 4 College of Life Science and Technology, Huazhong University of Science and Technology, Wuhan 430074, P. R. China

**Keywords:** Diselenide, Paclitaxel, Nanoparticles, Selective cytotoxicity, Molecular mechanism

## Abstract

Diselenide-containing paclitaxel nanoparticles (SePTX NPs) indicated selectivity of cytotoxicity between cancerous and normal cells in our previous work. Herein, the mechanism is revealed by molecular biology in detail. Cancer cells and normal cells were treated with the SePTX NPs and cell proliferation was measured using 3-(4, 5-dimethyl-2-thiazolyl)-2, 5-diphenyl-2-H-tetrazolium bromide (MTT) assay and cell morphology. Measurement of reactive oxygen species (ROS) levels and biochemical parameters were employed to monitor oxidative stress of the cells. JC-1 assay was used to detect the mitochondrial dysfunction of the cells. Terminal deoxynucleotidyl transferase-mediated dUTP nick end labeling (TUNEL) analysis was used to detect apoptosis of the cells. Immunofluorescence analysis and western blotting were employed to monitor changes in signaling pathway-related proteins. Compared with PTX, SePTX NPs has a good selectivity to cancer cells and can obviously induce the proliferation damage of cancer cells, but has no significant toxicity to normal cells, indicating that SePTX NPs has a specific killing effect on cancer cells. The results of mechanism research show that SePTX NPs can successfully inhibit the depolymerization of microtubules and induce cell cycle arrest, which is related to the upregulation of p53 and CyclinB1. Simultaneously, SePTX NPs can successfully induce oxidative stress, cause mitochondrial dysfunction, resulting in mitochondrial pathway-mediated apoptosis, which is related to the upregulation of autophagy-related protein LC3-II. On the other hand, lewis lung cancer C57BL/6 mice were used to evaluate the anti-tumor effects of SePTX NPs *in vivo*. Our data show that SePTX NPs exhibited high inhibiting efficiency against the growth of tumors and were able to reduce the side effects. Collectively, these data indicate that the high antitumor effect and selective cytotoxicities of SePTX NPs is promising in future cancer therapy.

## Introduction

Chemotherapy is one of the important strategies for treating solid cancers 1-4. Paclitaxel (PTX), a monomeric diterpene alkaloid compound extracted from the bark of Taxus chinensis, has been widely used as anticancer drug for a series of malignancies, including breast, endometrial, liver, bile duct and esophageal cancers 5-8. Patients generally show a favorable initial response to PTX, however, the efficacy of PTX is frequently restricted by the lack of tumor specificity. What's worse, increasing dose can cause a variety of severe side effects 9. Therefore, it is urgently needed to develop new drug formulations that can effectively improve the PTX tumor-specific efficacy and reduce the adverse reactions in cancer treatment. It is challenge to develop the PTX formulation with tumor selectivity. The most widely used method is conjugating the targeting molecules with PTX 10-17. Alternatively, the tumor microenvironment is utilized for enhancing the selectivity between normal and cancer cells 18-25.

Selenium can inhibit oxidative stress, DNA damage and apoptosis, and regulate cell proliferation and cell cycle 26, 27. Selenium deficiency is considered to be one of the risk factors for malignant tumors 28. Selenium supplement can effectively antagonize the development of malignant tumors 29, 30. In recent years, the anticancer efficacy of some selenium compounds has gained widespread attention in the field of cancer chemotherapy 31, 32. The formation of some selenium compounds is attributed to the special atomic radius and electronegativity of selenium. The bond energy of S-S is 240 kJ·mol^-1^, and the bond energy of C-C is 364 kJ·mol^-1^, the bond energy of Se-Se is 172 kJ·mol^-1^. It can be seen that the bond energy of Se-Se is smaller than the bond energy of S-S and C-C. This difference makes it more sensitive to be oxidized and reduced than low valence state sulfur compounds. In recent years, some selenium compounds with stimulating sensitivity and antioxidant activity have been reported in pharmacochemistry. For example, some amphiphilic polymers not only have different topologies, but also have a diselenide bond. When they are self-assembled into micelles, they can successfully become drug-delivered diselenide-containing nanoparticles 33-36. It is worth noting that the reports of organic molecules composing diselenide-containing nanoparticles are still rare.

In our previous work developed redox-hypersensitve diselenide-containing PTX nanoparticles (SePTX NPs) for selective treatment of cancer cells 37. The cellular proliferation inhibition toward cancer cells was significantly higher than that toward normal cells 37. The selectivity was ascribed to the increasing reactive oxygen species in cancer cells, but the molecular mechanism is unclear yet. In this work, the mechanism of inducing selective cytotoxicity towards cancer cells was investigated in detail. The aim of this study was to investigate the roles of intracellular oxidative stress, mitochondrial dysfunction, p53, CyclinB1 and autophagy, and their crosstalk in SePTX NPs-induced apoptosis pathways. At the same time, the* in vivo* investigations on SePTX NPs were also conducted using lewis lung carcinoma (LLC) tumor-bearing C57BL/6 mice to assess their antitumor efficacy.

## Materials and methods

### Materials

Mouse fibroblast L929 cells and human cervical cancer HeLa cells was obtained from the Shanghai Institute of Biochemistry and Cell Biology (Shanghai, China). The HeLa cells and L929 cells were cultured in dulbecco's modified eagle's medium (DMEM) culture medium supplemented with 10% fetal bovineserum (FBS) and antibiotics (penicillin 100 U·mL^-1^ and streptomycin 100 μg·mL^-1^) at 37^o^C in a 5% CO_2_ atmosphere. DMEM, FBS and collagenase type II were purchased from Gibco (LosAngeles, CA, USA). 3-(4,5-dimethyl-2-thiazolyl)-2,5-diphenyl-2-H-tetrazolium bromide (MTT) was purchased from Dojindo (Kumamoto, Japan). Reactive oxygen species (ROS) fluorescent probe-dihydroethidium (DHE) was obtained from vigorous (Vigorous Biotechnology, USA). Superoxide dismutase (SOD) assay kit was purchased from Dojindo Molecular Technologies (Dojindo, Japan). Malondialdehyde (MDA) assay kit was purchased from Nanjing Jiancheng Bioengineering Institute (Nanjing, China). JC-1 Mitochondrial membrane potential assays kit was obtained from Abnova (Taipei City, Taiwan). Terminal deoxynucleotidyl transferase-mediated dUTP nick end labeling (TUNEL) assay kit was obtained from Millipore (Billerica, MA). CyclinB1, P53, Bcl-2, Bax, Caspase-3, LC3, LC3-II and β-actin antibody were purchased from Abcam (Boston, MA, USA). The SePTX NPs were synthesized and donated by Changchun Institute of Applied Chemistry, Chinese Academy of Sciences. All reagents were purchased from sigma (St. Louis, MO, USA).

### Synthesis of SePTX NPs

The synthesis method of SePTX is based on previous reports 37. The nano-precipitation method was used to prepare NPs. First, 5 mL of tetrahydrofuran (THF) was prepared for dissolving 2 mg of SePTX. After stirring for 5 min, 10 mL of deionized water was prepared, and the stirred solution was dropped into deionized water while stirring, and the organic solvent was evaporated, and dialyzed for 24 h. High performance liquid chromatography (HPLC, Shimadzu, CBM-20A) was used to measure the concentration of SePTX NPs. The elution rate is 1.0 mL·min^-1^. The volume of the sample we injected is 20 μL, and the dilution is 10 times. The mobile phase used is a mixed liquid of methanol/acetonitrile/ water with a mixing ratio of 42.5/42.5/15 (v/v/v). The wavelength of SePTX was detected to be 239 nm. The concentrations of SePTX NPs were determined by HPLC and UV-vis spectrophotometer mentioned above. Dynamic light scattering (DLS) was used to measure the zeta potential and particle size of SePTX NPs. Transmission electron microscopy (TEM) was used to measure the morphology of SePTX NPs.

### Cell culture

The human cervical cancer HeLa cell line and human breast adenocarcinoma MCF7 cell line and the mice fibroblast L929 cell line and human bronchial epithelial BEAS-2B cell line were routinely grown in DMEM containing 10% FBS and antibiotics (penicillin 100 U·mL^-1^ and streptomycin 100 μg·mL^-1^). All the cells were cultured in a humidified incubator at 37^o^C with 5% CO_2_.

### Cytotoxicity and cell morphology

The cell proliferation assay uses the MTT method. A 96-well plate was prepared and cells were seeded, and the number of cells per well reached 1×10^4^ cells. Different concentrations of PTX or SePTX NPs were added to each well and incubated for 48 h or 72 h. After the incubation, 20 μL of MTT (5 mg·mL^-1^ in PBS) solution was added to each corresponding well and incubation was continued for 4 h at 37^o^C. The supernatant was discarded and 150 μL of DMSO was added to each well. A microplate reader (Perkinelmer Inc., Waltham, MA, USA) was used to detect the absorbance of each well at a wavelength of 490 nm.

In order to observe the morphological changes of the cells, cells were seeded in a 6-well plate, and the number of cells per well reached 1×10^4^ cells, and incubated for 24 h. After the cells were completely adhered, the cells were washed with PBS (pH 7.4), and blank medium or SePTX NPs were added to each corresponding well, and incubation was continued for 48 h. After the 48 h, the supernatant was aspirated, and the cells were washed with PBS (pH 7.4), and 500 μL of PBS (pH 7.4) was added. An Olympus IX71 microscope (Olympus Corporation, Tokyo, Japan) was used to observe the morphological changes of each group of cells.

### Measurement of ROS levels

The ROS fluorescent probe-DHE (Vigorous Biotechnology, USA) was used to measure the level of intracellular ROS. The coverslips were placed in a six-well plate and cells were seeded on the coverslips and incubated at 37^o^C for 24 h. After the cells were attached, the supernatant was aspirated and blank medium or SePTX was added to each corresponding well, and incubation was continued for 48 h. After the 48 h, the supernatant was aspirated, and the cells were washed with PBS (pH 7.4), and 10 μM of DHE was added to each well. After incubation for 20 min, the supernatant was aspirated, and the cells were washed with PBS (pH 7.4). After that, 4', 6-diamidino-2-phenylindole (DAPI) nucleus staining was performed using the same method described above. Take out the coverslip and rapidly mounted for observation. Fluorescence microscopy (Olympus IX71, Japan) is used to detect fluorescent intensity (red, ROS; and blue, DAPI) in cells and quantitative analysis using image analysis software.

### Analysis of biochemical parameters

The thiobarbituric acid reactant method is used to measure the activity of MDA. A xanthine/ xanthine oxidase system produces O^2-^•, the assay of SOD activity was based on its ability to inhibit the oxidation of oxymine by O^2-^•. An automated microplate reader (Biotek Synergy 2, VT, USA) was used for colorimetric analysis. Coomassie blue staining is used to measure protein concentration. Detailed experimental procedures need to be in accordance with the instructions for each kits.

### JC-1 assays

JC-1 assay kit (Abnova, Taipei City, Taiwan) was used for JC-1 analysis. The coverslips were placed in a six-well plate and cells were seeded on the coverslips and incubated at 37^o^C for 24 h. After the cells were attached, the supernatant was aspirated and blank medium or SePTX was added to each corresponding well, and incubation was continued for 48 h. After the 48 h, the supernatant was aspirated, and the cells were washed with PBS (pH 7.4), and 10 μM of JC-1 was added to each well. After incubation for 20 min, the supernatant was aspirated, and the cells were washed with PBS (pH 7.4). Take out the coverslip and rapidly mounted for observation. Fluorescence microscopy (Olympus IX71, Japan) is used to detect fluorescent signals in cells and quantitative analysis using image analysis software.

### TUNEL analysis

TUNEL detection kit (Millipore, Billerica, MA) was used for TUNEL analysis. The coverslips were placed in a six-well plate and cells were seeded on the coverslips and incubated at 37^o^C for 24 h. After the cells were attached, the supernatant was aspirated and blank medium or SePTX was added to each corresponding well, and incubation was continued for 48 h. After the 48 h, the supernatant was aspirated, and the cells were washed with PBS (pH 7.4). Detailed operation of TUNEL staining according to the manufacturer's instructions. After the staining of TUNEL, the supernatant was aspirated, and the cells were washed with PBS (pH 7.4). After that, DAPI nucleus staining was performed using the same method described above. Take out the coverslip and rapidly mounted for observation. Fluorescence microscopy (Olympus IX71, Japan) is used to detect fluorescent intensity (red, TUNEL; and blue, DAPI) in cells and quantitative analysis using image analysis software.

### Immunofluorescence analysis

After incubation with SePTX NPs for 48 h, the cells were incubated in 4% paraformaldehyde for 20 min, 1% of bovine serum albumin was used for cell blocking, p53 (ab78316; Abcam) or LC3-II (ab48394; Abcam) was used for incubation of primary antibodies. The blocking time was 30 min at room temperature and the primary antibody was incubated overnight at 4^o^C. Cy3-labeled goat anti-rabbit IgG (A0516; Beyotime) was used for incubation of the secondary antibody, and the secondary antibody were incubated for 1.5 h at 4^o^C. DAPI was used for staining of nuclei, and confocal microscopy (Carl Zeiss LSM780; Instrument Development Center, NCKU, Tainan City, Taiwan) was used for observation of intracellular fluorescence intensity.

### Western blot analysis

Western blot was used to analyze the expression levels of various proteins in cells treated with SePTX NPs. SePTX NPs-treated cells were subjected to cell lysis and protein quantification to prepare the western blot samples. The bicinchoninic acid protein assay reagent (Pierce, Rockford, IL, US) was used for quantitative analysis of proteins. Sodium dodecyl sulfate-polyacrylamide gel electrophoresis analysis was used for electrophoretic separation of proteins. After electrophoresis separation, trarsmembran, membrane blocking, primary antibody incubation, and secondary antibody incubation were performed separately. Finally, enhanced chemiluminescence kit (Amersham Biosciences, Piscataway, NJ) is used to detect protein bands. The primary antibodies were used: CyclinB1 (ab32053; Abcam), p53 (ab78316; Abcam), LC3 (ab51520; Abcam), BAX (ab32503; Abcam), Bcl-2 (ab32124; Abcam), Caspase-3 (ab4051; Abcam), and β-actin (ab8227; Abcam).

### Antitumor efficacy

LLC tumor-bearing C57BL/6 mice were randomly divided into 3 groups: (1) control group (normal saline), (2) PTX group and (3) SePTX NPs group. Before injection, all mice were marked and weighed, and the length and width of tumors were measured to determine their initial volume. The day of starting injection was designated as day 1. For groups (2) and (3), an equivalent PTX dose of 15 mg/kg body was intravenously injected via tail vein on day 1, day 3 and day 5, separately. For group (1), the mice were injected with an equivalent volume of normal saline. The tumor size was measured every other day, and its volume calculated. The mouse survival rate in each group was recorded daily. The tumor size was measured every other day, and the tumor volume was estimated by the following formula:

V (mm3) = [length × width2] / 2 (1)

### Statistical analysis

The mean±SEM is used to represent the data obtained from the experiment. The Student-Newman- Keuls test in one-way analysis was used for statistical analysis. Differences were considered to be significant at *p*<0.05.

## Results

### Preparation of SePTX NPs

**Figure 1:**
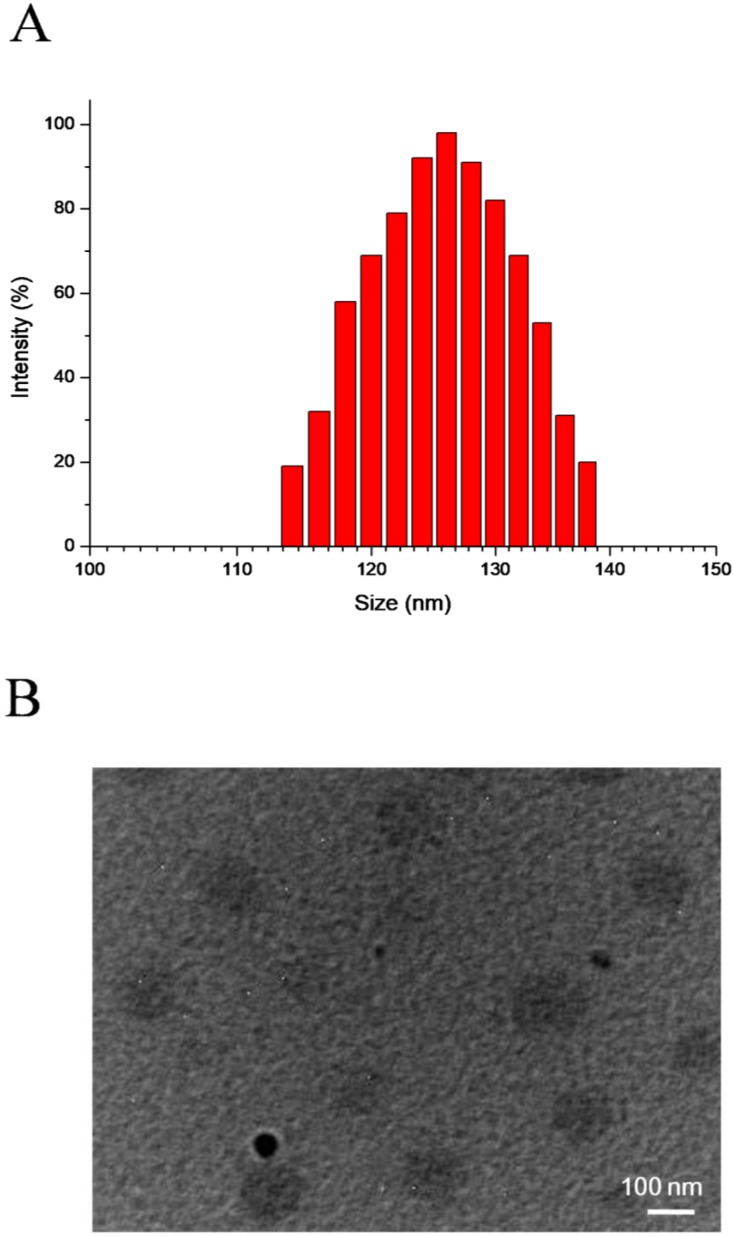
DLS results (A) and TEM image (B) of SePTX NPs.

SePTX NPs are formed by self-assembly of diselenide-containing PTX in aqueous solution. DLS and TEM were used to detect the particle size distribution and morphology of SePTX NPs, respectively. The particle size and polydispersity index of SePTX NPs detected by DLS were 126 nm and 0.14, respectively (Figure 1A). As shown in Figure 1B, the TEM image shows that SePTX NPs are uniformly dispersed spherical morphology whose particle size is close to the size measured by DLS.

### Cytotoxicity of SePTX NPs on cancer cells

**Figure 2:**
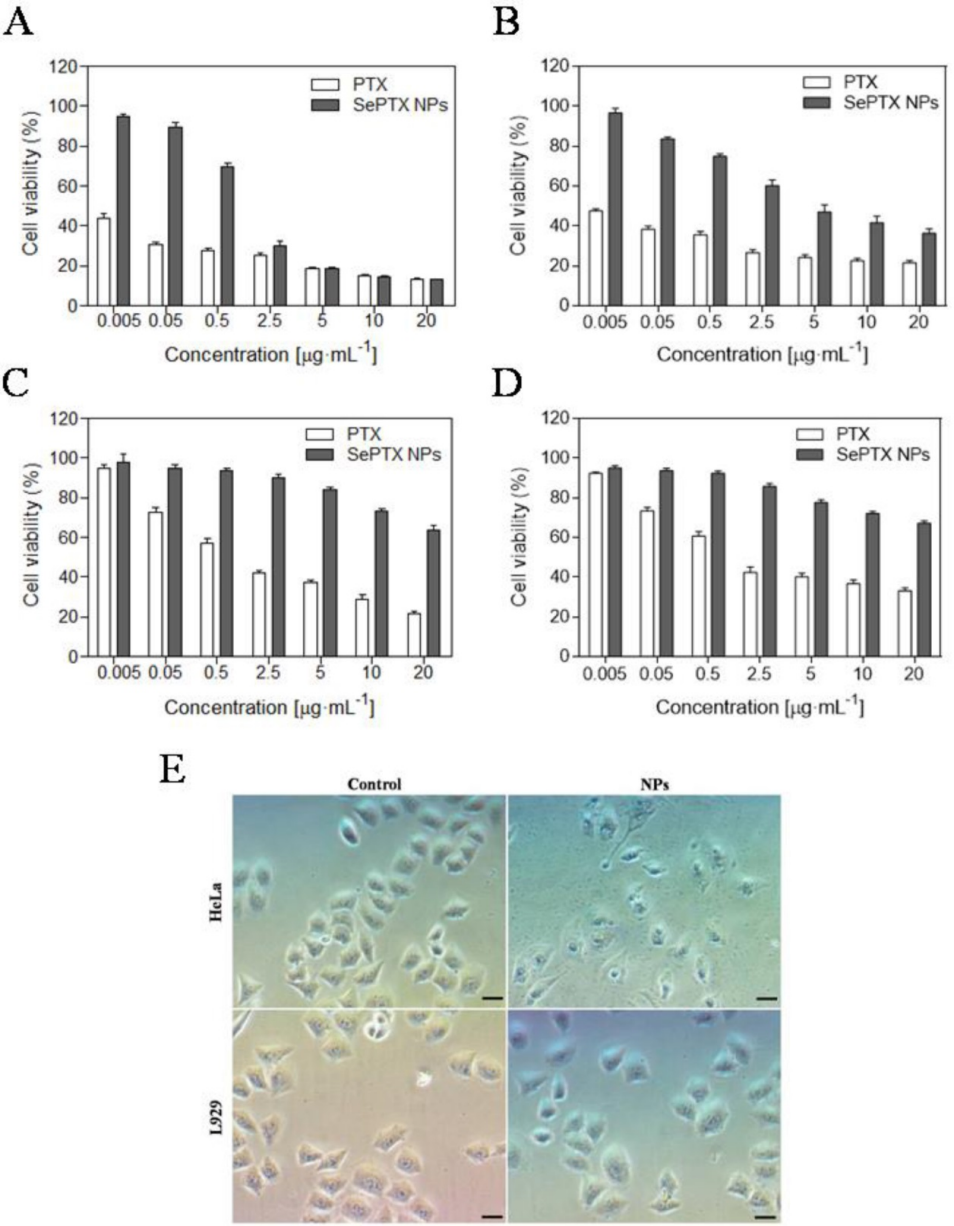
***In vitro* cytotoxicities of different concentrations of PTX and SePTX NPs toward (A)** HeLa cells, **(B)** MCF7 cells, **(C)** L929 cells and **(D)** BEAS-2B cells incubated for 48 h. Effect of SePTX NPs on cell morphology **(E)**. Phase contrast microscopy image of HeLa cells and L929 cells without and with SePTX NPs treatment for 48 h, respectively. Scale bars=50 μm.

The MTT assay is used to detect the toxicity of SePTX NPs or free PTX to cancer cells and normal cells. As shown in Figure 2, SePTX NPs are less toxic to cells than free PTX. This is because free PTX and SePTX NPs enter the cell using a different mechanism, that is, PTX is rapidly diffused into the cell, and SePTX NPs enter the cell by endocytosis. More interestingly, SePTX NPs showed a strong cytotoxicity to cancer cells (containing HeLa cells or MCF7 cells), but toxicity to normal cells (containing L929 cells or BEAS-2B cells) was significantly weaker. More than 80% of HeLa cells and 76% of MCF7 cells were killed at the concentration of 5 μg·mL^-1^ of SePTX NPs (Figure 2A and Figure 2B). In contrast, less than 20% of L929 cells and 22.5% of BEAS-2B cells were killed at 5 μg·mL^-1^ of SePTX NPs, as shown in Figure 2C and Figure 2D. The IC_50_ values were about 0.79 μg·mL^-1^ and 2.98 μg·mL^-1^ toward HeLa cells and MCF7 cells, which were actually much smaller than that of the normal cells. These results indicated the SePTX NPs possessed good selectivity on cancer cells.

Subsequently, we investigate the effects of SePTX NPs on the morphology of normal cells and cancer cells. Cell morphology can reflect the apoptotic state of cells. All cells are treated with SePTX NPs (PTX: 5 μg·mL^-1^). As shown in Figure 2C, both L929 cells and HeLa cells show polygonal morphology and the shape of normal cell edge in the absence of SePTX NPs. However, after being treating with SePTX NPs, L929 cells did not show prominent cell damage-related morphological changes. HeLa cells displayed outstanding apoptotic morphological changes, such as cell membrane atrophy, suppression, chromatin thickening, and eventual fragmentation, which further developed into cytoplasmic membrane dividing the cytoplasm to surround the cytoplasm and nuclear fragments, finally forming the apoptotic bodies. SePTX NPs can cause obvious morphological changes in cancer cells, demonstrating the specific killing effect of SePTX NPs on cancer cells.

### SePTX NPs increased ROS generation, increased MDA content, and reduced SOD activity in cancer cells

**Figure 3:**
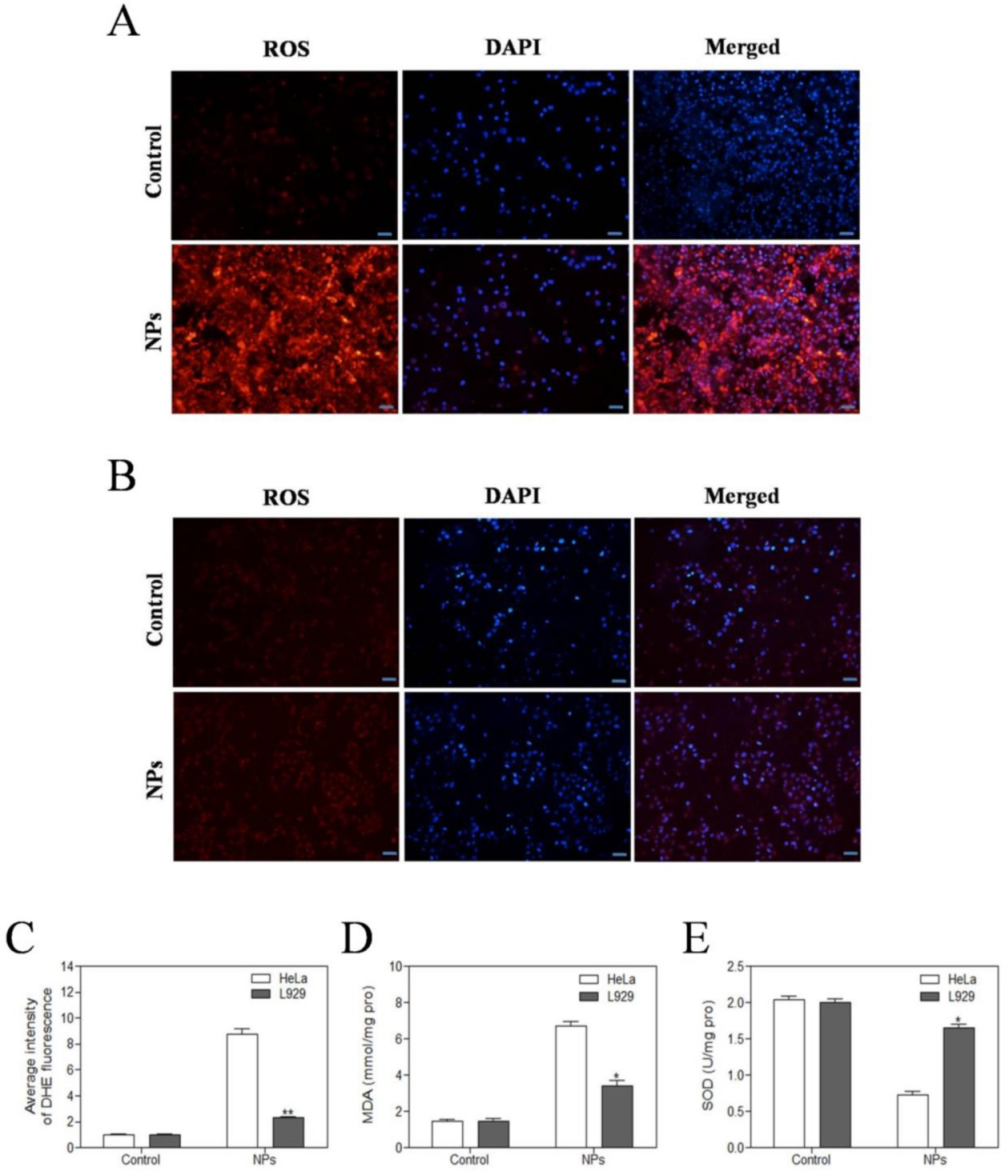
** Evaluation of oxidative stress levels in HeLa cells (A)** and L929 cells **(B)** after SePTX NPs treatment. Intracellular ROS levels were measured with fluorescence imaging using the DHE probe in cells cultured in the presence of SePTX NPs (PTX: 5 μg·mL^-1^) for 48 h. Scale bars=20 μm. **(C)** The average intensity of DHE fluorescence in L929 cells and HeLa cells. **(D)** The MDA level in L929 cells and HeLa cells. **(E)** The SOD level in L929 cells and HeLa cells. n=3; *p<0.05 *vs* HeLa group, **p<0.01 *vs* HeLa group.

The level of intracellular ROS was quantified by DHE fluorescence intensity. DHE fluorescent probes can freely pass through the cell membrane and are oxidized to emit red fluorescence by ROS in the cells. The red fluorescence intensity of SePTX NPs (PTX: 5 μg·mL^-1^) group in HeLa cells was significantly enhanced (Figure 3A). Compared with HeLa cells, almost no red fluorescence is visible for L929 cells at the same SePTX NPs concentration (Figure 3B), confirming that SePTX NPs could significantly increase the level of ROS in the cancer cells, but had no significant effect on the level of ROS in normal cells (Figure 3C). Similarly, SePTX NPs could significantly increase the level of MDA in cancer cells and reduce the content of SOD in cancer cells, but had no obvious effect on MDA and SOD in normal cells (Figure 3D and Figure 3E).

### SePTX NPs-induced mitochondrial damage in cancer cells

**Figure 4:**
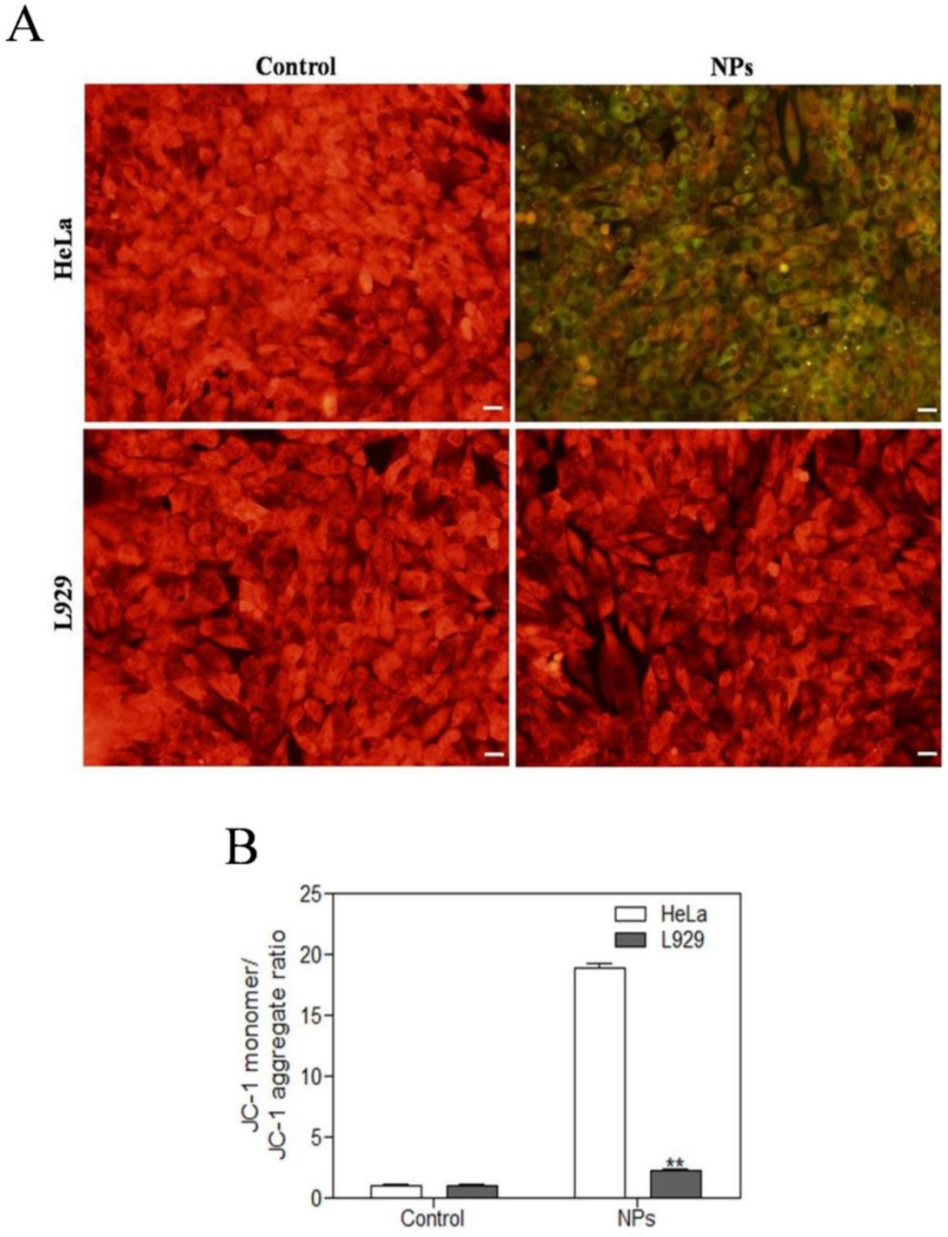
** Effects of SePTX NPs on mitochondrial membrane permeability in HeLa cells and L929 cells**. Cells were treated with SePTX NPs (PTX: 5 μg·mL^-1^) for 48 h. **(A)** Mitochondrial membrane potential (Δψm) was evaluated using JC-1 in treated cells. Red fluorescence indicates JC-1 aggregates within the mitochondria in cells, whereas green fluorescence indicates JC-1 monomers in the cytoplasm and loss of Δψm. Scale bars=20 μm. **(B)** Ratio of JC-1 monomers to JC-1 aggregated. n=3; **p<0.01 *vs* HeLa group.

JC-1 assay was used to detect the mitochondrial dysfunction of the cells. The normal cells could maintain intact functional activity of mitochondria, which show red JC-1 mitochondrial membrane potential aggregates, while the damaged mitochondria are in green with enhanced JC-1 monomers. As shown in Figure 4, SePTX NPs (PTX: 5 μg·mL^-1^) treated cancer cells showed a significant changes in JC-1 fluorescence from red to green. While, there is no obvious changes of JC-1 fluorescence from red to green for the SePTX NPs treated normal cells.

### SePTX NPs-induced apoptosis in cancer cells

**Figure 5:**
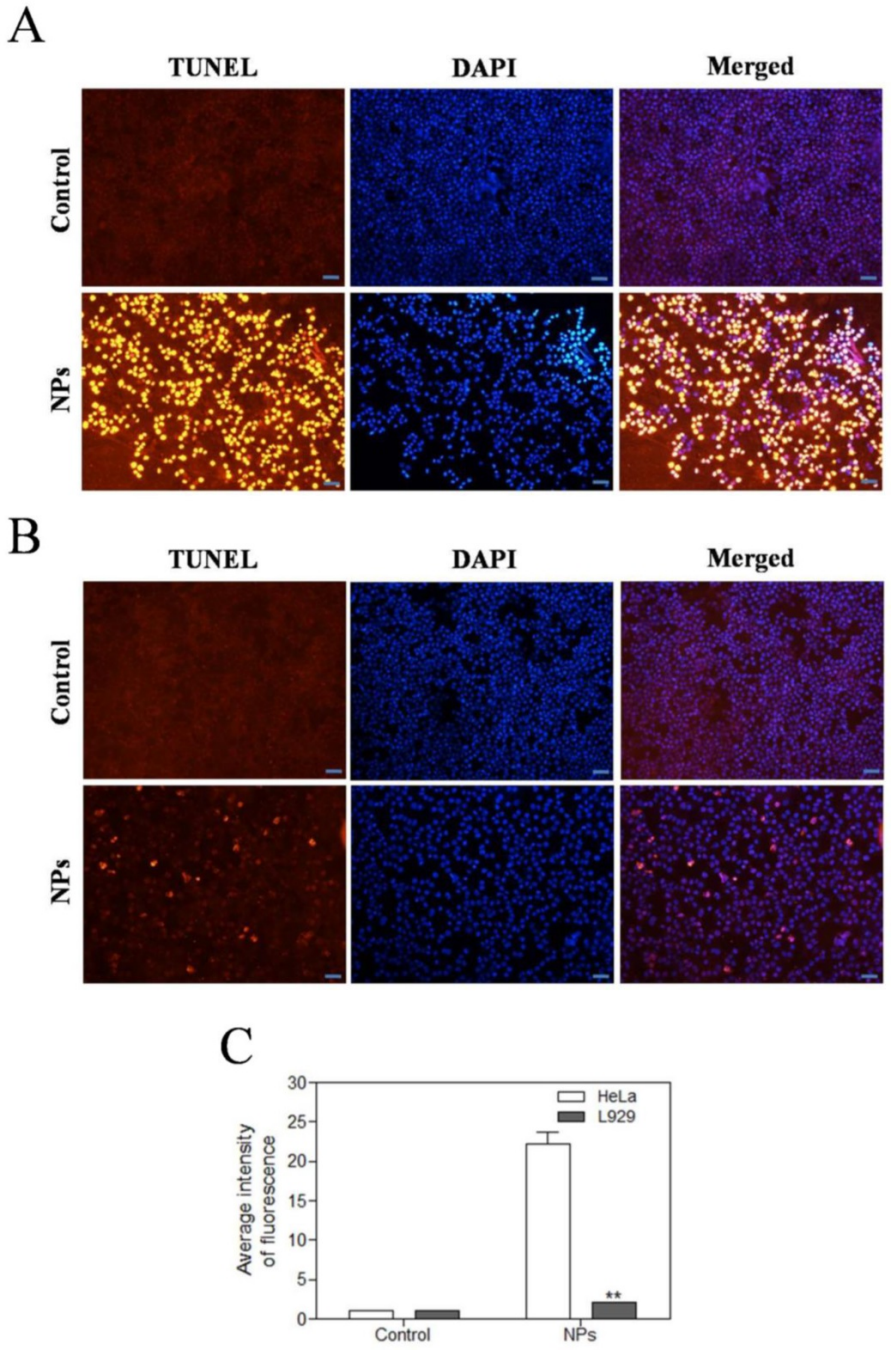
** Effects of SePTX NPs on apoptosis in HeLa cells (A)** and L929 cells **(B)**. Cells were treated with SePTX NPs (PTX: 5 μg·mL^-1^) for 48 h. Apoptosis was assessed in a TUNEL assay; the nuclei were counterstained with DAPI. Representative images show apoptotic (fragmented) DNA (red staining). Scale bars=20 μm. **(C)** The average intensity of TUNEL fluorescence in HeLa cells and L929 cells. n=3; **p<0.01 *vs* HeLa group.

We further investigated the effects of SePTX NPs on apoptosis of tumor and normal cells by using TUNEL assay. The red fluorescence intensity of SePTX NPs (PTX: 5 μg·mL^-1^) group in HeLa cells was significantly enhanced (Figure 5A). Compared with HeLa cells, almost no red fluorescence is visible for L929 cells at the same SePTX NPs concentration (Figure 5B). Figure 5C clearly reveals that SePTX NPs-treated cancer cells possess higher levels of TUNEL positive cells than normal cells.

### SePTX NPs-induced inhibition of microtubule depolymerization in cancer cells

**Figure 6:**
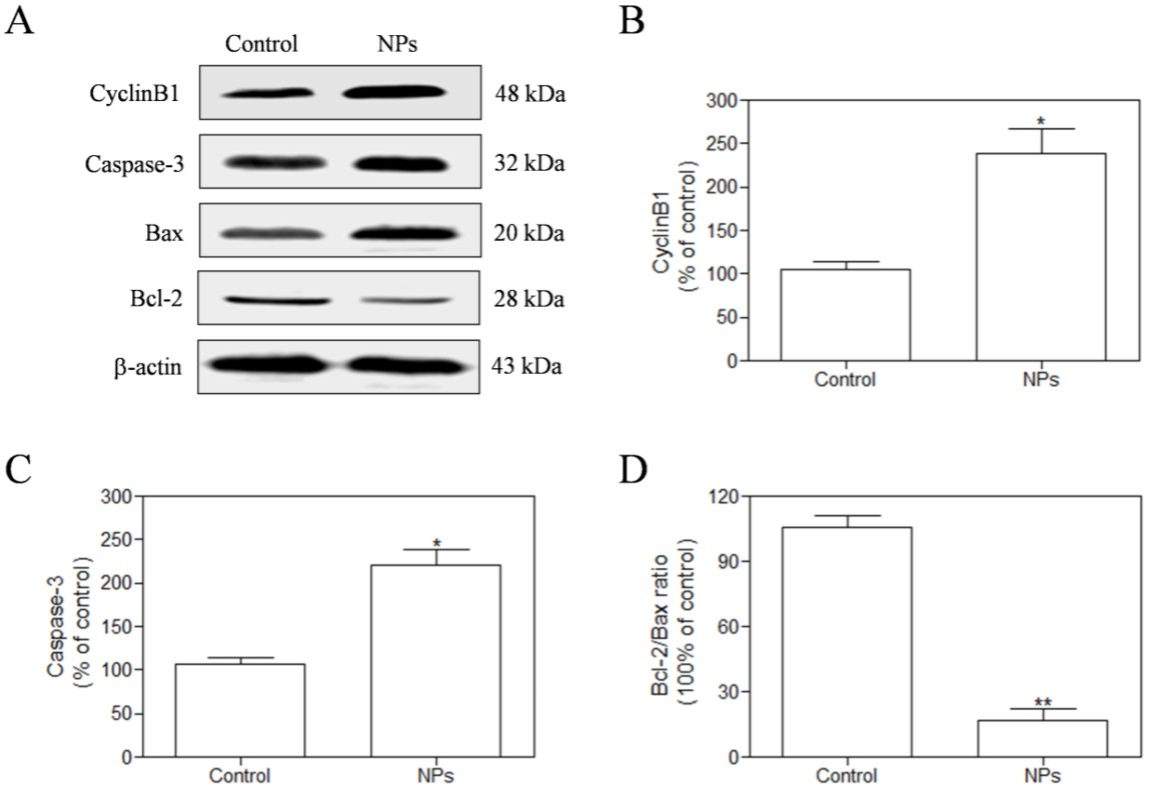
** The HeLa cells were treated with SePTX NPs (PTX: 5 μg·m^L-1^) for 48 h and the expression analysis of CyclinB1,** Caspase-3, Bax, and Bcl-2 was performed by western blot analysis. **(A)** Representative bands of CyclinB1, Caspase-3, Bax, and Bcl-2 (inner reference: β-actin). **(B)** Quantitative determination of CyclinB1/β-actin ratio. **(C)** Quantitative determination of Caspase-3/β-actin ratio. **(D)** Quantitative determination of Bcl-2/Bax ratio. n=3; *p<0.05 *vs* control group, **p<0.01 *vs* control group.

We measured the expression of CyclinB1 by western blotting assay in the HeLa cells after exposure to SePTX NPs (PTX: 5 μg·mL^-1^) for 48 h. Our findings demonstrate that the expression of CyclinB1 in HeLa cells is up-regulated by SePTX NPs (Figure 6A and Figure 6B). Expression of mitochondrial pathway apoptosis-related proteins, such as Caspase-3, Bax and Bcl-2, was detected using western blotting analysis. Our findings demonstrate that, the expression of Caspase-3 and Bax was up-regulated after treatment with SePTX NPs (Figure 6A and Figure 6C). Considering a fact that the Bcl-2 protein is indicative of the resistance to cell apoptosis while the Bax protein is associated with the occurrence of cell apoptosis, a right ratio between Bcl-2 and Bax is a crucial factor for cells to survive 38. It can be seen from Figure 6D that the Bcl-2/Bax ratio for the SePTX NPs-treated cells was about 5-folds lower than that for control, implying that these cells underwent apoptosis.

### SePTX NPs-induced overexpression of p53 protein in cancer cells

**Figure 7:**
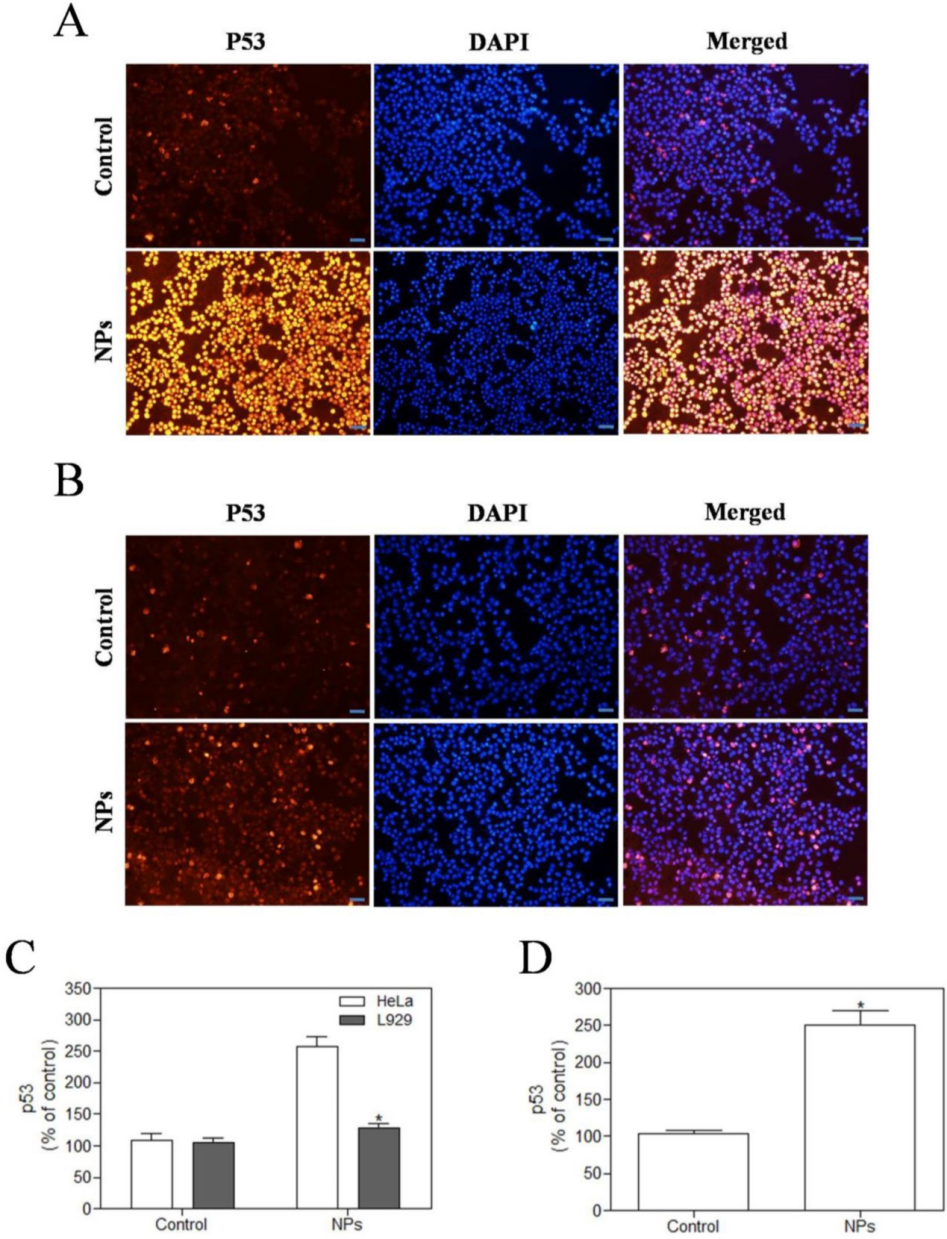
** SePTX NPs exposure increases apoptosis in HeLa cells and L929 cells in 48 h. HeLa cells (A)** and L929 cells **(B)** were treated with SePTX NPs (PTX: 5 μg·mL^-1^) for 48 h and then processed for immunofluorescence analysis, Scale bars=20 μm; **(C)** the average intensity of p53 fluorescence in HeLa cells and L929 cells. n=3; *p<0.05 *vs* HeLa group. **(D)** The expression analysis of p53 in HeLa cells was performed by western blot analysis. n=3; *p<0.05 *vs* control group.

We used immunofluorescence analysis to detect expression of p53. These results showed that SePTX NPs (PTX: 5 μg·mL^-1^) could induce the expression of cell cycle related critical tumor suppressor protein p53 on cancer cells (Figure 7A). However, in normal cells, SePTX NPs did not significantly induce the expression of p53 protein (Figure 7B), which suggests that the SePTX NPs-induced p53 protein expression has a specific effect on cancer cells. The quantitative analysis of the expression of p53 in cancer cells and normal cells was shown in Figure 7C. Next, we used western blot analysis to detect expression of p53. This study found that p53 protein level in Hela cells treated by SePTX NPs was up-regulated significantly, which is also consistent with immunofluorescence data (Figure 7D).

### SePTX NPs-induced autophagy in cancer cells

**Figure 8:**
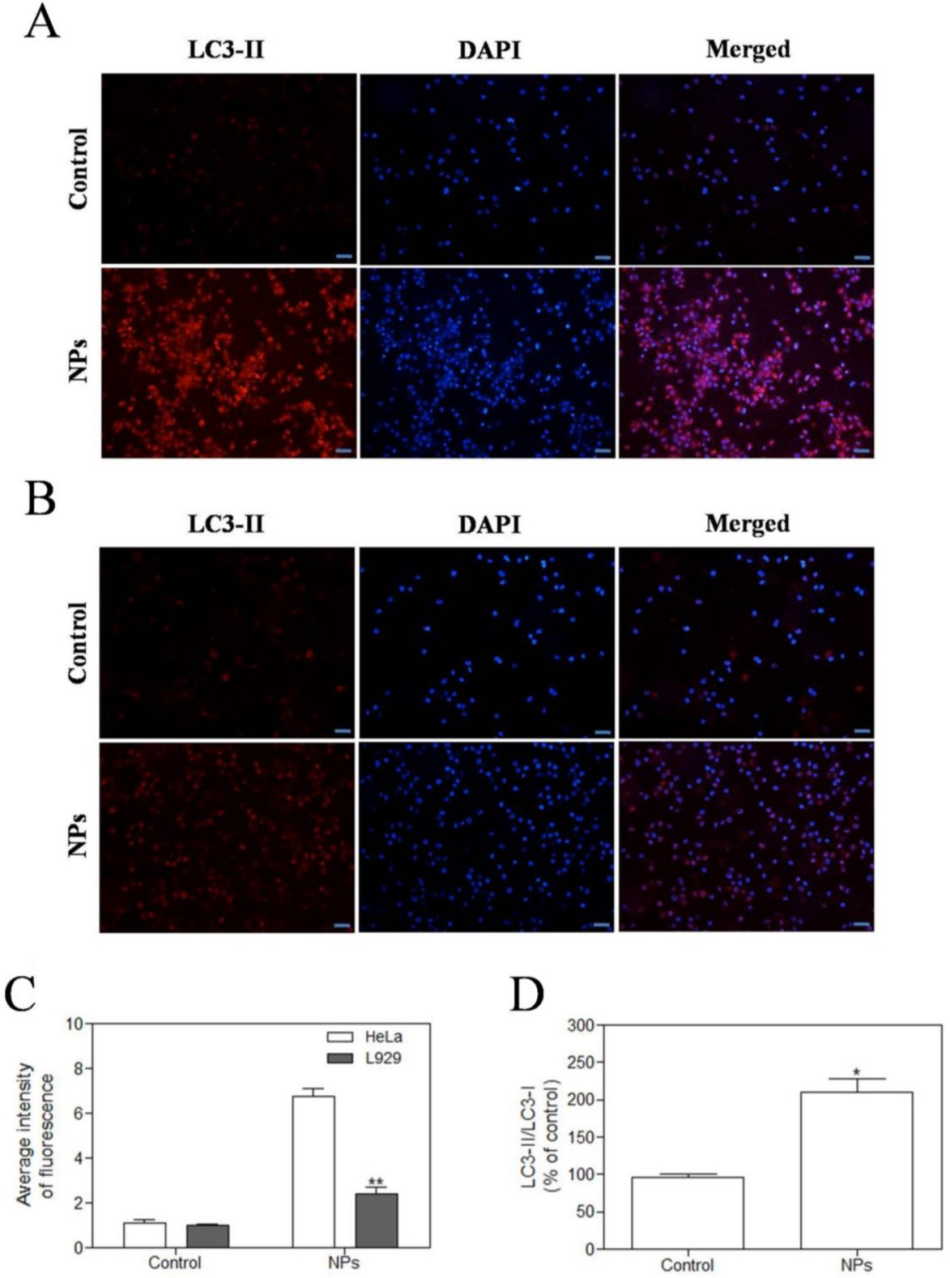
** SePTX NPs exposure increases autophagy in cultured HeLa cells and L929 cells in 48 h.** HeLa cells **(A)** and L929 cells **(B)** were treated with SePTX NPs (PTX: 5 μg·mL^-1^) for 48 h and then processed for immunofluorescence analysis, Scale bars=20 μm; **(C)** The average intensity of LC3-II fluorescence in HeLa cells and L929 cells. n=3; **p<0.01 *vs* HeLa group. **(D)** The ratio analysis of LC3-II/LC3-I was performed by western blot analysis. n=3; * *p*<0.05 *vs* control group.

In the study, we observed the effect of SePTX NPs on the expression of autophagy-associated protein LC3-II in HeLa cells and L929 cells. Immunofluorescence analysis was employed to monitor changes in LC3-II proteins. Red fluorescent labeled LC3-II antibody was used to detect the level of autophagy and DAPI was used to localize the cells. These results showed that SePTX NPs (PTX: 5 μg·mL^-1^) could induce the expression of LC3-II on cancer cells (Figure 8A). However, in normal cells, SePTX NPs did not significantly induce the expression of LC3-II protein (Figure 8B), which suggests that the SePTX NPs-induced LC3-II protein expression has a specific effect on cancer cells. The quantitative analysis of the expression of LC3-II in cancer cells and normal cells was shown in Figure 8C. This result suggests that SePTX NPs can effectively activate the autophagy of cancer cells so as to promote the apoptosis of cancer cells, but have not obvious effects in normal cells. Next, we used western blot analysis to detect expression of autophagy related protein LC3-I and LC3-II. This study found that ratio of LC3-II/LC3-I in Hela cells treated by SePTX NPs was up-regulated significantly (Figure 8D).

### *In vivo* antitumor efficacy

**Figure 9:**
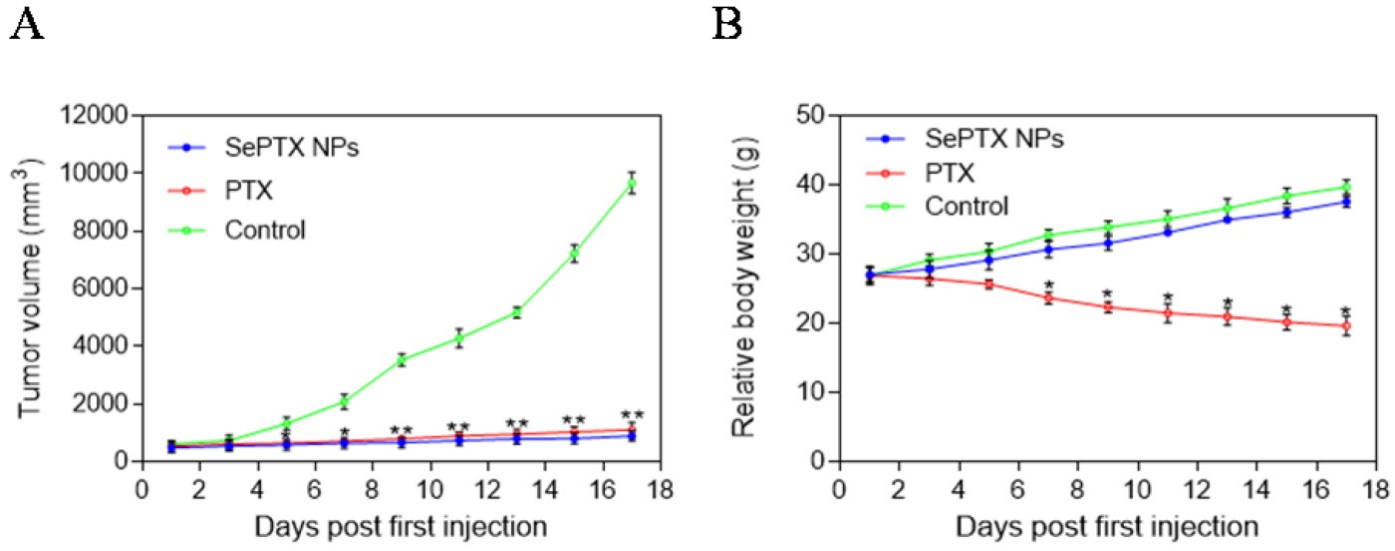
** Variations in tumor size with time after administration of different agents and changes in body weight of mice. (A)** Variations in tumor size with time after administration of PTX and SePTX NPs. **(B)** Changes in body weight of LLC tumor-bearing mice. n=5; * *p*<0.05 *vs* control group; ** *p*<0.01 *vs* control group.

Antitumor effects of different PTX formulations were evaluated using LLC tumor-bearing C57BL/6 mice. The variation of tumor volume with time was shown in Figure 9A. It was observed that the tumor volume of mice in control group rapidly increased starting from day 5; after injection of PTX alone, tumor volume of mice significantly reduced when compared to that in the control group. As shown in Fig. 9A, it is interesting that SePTX NPs have obvious anti-tumor effect and are similar to free PTX. The changes in body weight of mice were also monitored, and relevant data are depicted in Figure 9B. Among all test groups, PTX group mice showed serious weight loss, due to the highest systemic toxicity. In SePTX NPs group, mice had little weight loss and even gained the body weight constantly because of the lower systemic toxicity.

## Discussion

The development of a less toxic drug with selective targeting of cancer cells is very valuable in tumor research, and this can be achieved by nanomedicine 39-41. Specifically, the surface of the nanocarrier is modified by some substances such as a small molecule (galactose or folic acid) 10-12, a peptide (RGD) 13, a short oligonucleotide (aptamer) 14-17. The attachment of the above substances to the nanocarriers can exert tumor selective targeting due to several characteristics of tumor cells and tumor tissues, for example, tissue hypoxia, special biological enzymes, higher reduction potential and ROS levels (oxidative stress), lower pH values, etc 42-44. Therefore, the concept of "smart nanocarriers" has gradually been proposed, it is a strategy that can show rapid responses to enzymes 18, 19, high concentrations of glutathione 20, 21, and lower pH value 22-25 in the tumor microenvironment. In the present work, reduction-sensitive diselenide-containing antitumor drug SePTX were synthesized, and these molecules could self-assemble into NPs. It can be seen from basic parameters of SePTX NPs that the SePTX NPs had small sizes, suggesting that they have potential for practical applications. Cytotoxicities of SePTX NPs toward the cancer cells and the normal cells were evaluated by MTT assays and morphological changes. Our results suggest that SePTX NPs is less toxic to normal cells but shows strong cytotoxicity on cancer cells. However, after being treating with SePTX NPs, L929 cells did not show prominent cell damage-related morphological changes. SePTX NPs can cause obvious morphological changes in cancer cells, demonstrating the specific killing effect of SePTX NPs on cancer cells.

When the tumor tissues are exposed to oxidative stress, the amount of ROS in tumor tissues and cells is relatively higher than that can be scavenged by the body, which will result in the increase in lipid peroxidation level in tumor tissues, and induce DNA oxidative damage and cancer cell apoptosis 45. SOD is one of the important antioxidant enzymes maintaining the free radical balance in human body 46. The body can generate free radicals through the enzymatic and non-enzymatic systems, which can attack the polyunsaturated fatty acids in the membrane, trigger lipid peroxidation, and thus form lipid peroxides, such as MDA, ketones and hydroxyls 47. MDA is the preferred indicator to evaluate the ROS content *in vivo* 48. It has been reported that the role of ROS is increasingly gaining attention in the chemotherapy mechanisms of tumors 49. Studies have shown that, PTX can affect the activities of SOD and MDA in cancer cells 50, which will result in the severely imbalanced antioxidant capacity in cancer cells as well as the markedly elevated ROS level, eventually killing the cancer cells. Some recent studies have reported that various forms of Se can react with the reducing glutathione to produce ROS, which can thereby trigger some apoptotic signaling pathways and promote cancer cell apoptosis 51. Moreover, our data demonstrate that SePTX NPs shows a specific killing effect on cancer cells. Therefore, we believe that, compared with free PTX, the SePTX NPs may induce a higher level of cancer cell-specific oxidative stress, specifically induce cancer cell apoptosis, and thus play a synergistic role in promoting apoptosis. Our results showed that SePTX NPs can selectively induce oxidative stress in cancer cells, which may be related to mitochondrial dysfunction, thereby promoting the apoptosis of cancer cells.

In general, the potential source of ROS such as superoxide or hydrogen peroxide is derived from the mitochondrial respiratory chain 52. Mitochondrial permeability transition is regulated by a variety of factors, including reactive oxygen 52. PTX could induce mitochondrial damage, especially in mitochondrial membranes 53. Recent studies also show that selenized compound could induce apoptosis of A-375 cells by activating the mitochondrial pathway 54. Therefore, we hypothesized that selenized PTX may exert a stronger anti-tumor effect than free PTX, which may be related to ROS-mediated mitochondrial dysfunction. We tested the effect of SePTX NPs on the mitochondrial phosphate transporter regulation of tumor and normal cells. Our results suggests that SePTX NPs can specifically give rise to significant mitochondrial damage in cancer cells, and no significant effect on the mitochondrial function of normal cells. The results of TUNEL assay clearly reveal that SePTX NPs-treated cancer cells possess higher levels of cell apoptosis than normal cells. Combining the above results, SePTX NPs can specifically induce oxidative stress in cancer cells, higher levels of ROS can easily lead to mitochondrial dysfunction, which further causing cancer cell apoptosis.

PTX can induce stable tubulin polymerization and restrain the depolymerization 55. As a result, the vascular bundle cannot connect with the microtubule organizing center, which will block the cell cycle in G_2_/M phase, result in abnormal mitosis or arrest, and prevent from continuous division and cell death 55. CyclinB1 is an important cyclin in G2/M phase, which can form the complex with cyclin-dependent kinase 1 (CDK1), the mitosis promoting factor (MPF) 56. MPF can increase the number of cells transforming from G_2_ phase to M phase, and CyclinB1 in MPF protein will be rapidly degraded 56. It is found that PTX can prevent the degradation of CyclinB1 by means of ubiquitination of proteasome, which will give rise to prolonged CyclinB1/CDK1 activity 57, cell arrest in G_2_/M phase, and activation of apoptotic signal transduction pathway, and apoptosis 58. Such findings suggest that PTX-induced CyclinB1 expression is an important prerequisite to induce cancer cell apoptosis. Moreover, the selenium can also inhibit the depolymerization of tubulins from different pathways 59, interfere with the mitosis of cancer cells, and eventually lead to apoptosis of cancer cells 60. Therefore, we hypothesized that selenized PTX may exert a stronger anti-tumor effect than free PTX, which may be related to CyclinB1-mediated apoptosis. Our study finds that SePTX NPs acts as a potent microtubule depolymerization inhibitor significantly increased the expression of CyclinB1 in cancer cells, which can induce G_2_/M arrest, thus blocking cancer cell replication. Expression of mitochondrial pathway apoptosis-related proteins, such as Bax, Bcl-2, and Caspase-3, was detected using western blotting analysis. The results indicate that SePTX NPs can successfully induce cell cycle arrest and further induce mitochondria-mediated apoptosis.

The main physiological function of p53 protein is to acts on the cell cycle 61, 62. It can regulate the G_0_/G_1_ and G_2_/M period to block B51, and activate apoptosis 55. It was reported that p53 is a microtubule-associated protein that monitors the status of micro-tubes directly 63. Although PTX could not induce genetic damage, it can induce formation of aneuploidy and triggers p53 response, stabilizes microtubules by promoting the distribution of microtubules, then arresting the cell cycle at G_0_/G_1_ and G_2_/M period 64. Studies have shown that selenocysteine inhibits the depolymerization of microtubular proteins during mitosis anaphase and interferes with the mitosis of leukemia cells 47. It can be interpreted from that, PTX and selenium can inhibit the mitoses of cancer cells from different pathways, and eventually induce apoptosis of cancer cells. Therefore, in this study, we examined the expression of critical cell-cycle related tumor suppressor protein p53, and our experimental results showed that SePTX NPs resulted in a significant increase in p53 positive expression on cancer cells. However, in normal cells, SePTX NPs did not significantly induce the expression of p53 protein, which suggests that the expression of SePTX NPs on p53 protein has a specific effect on cancer cells. Next, western blot analysis found that p53 protein level in Hela cells treated by SePTX NPs was up-regulated significantly, which is also consistent with immunofluorescence data.

Previous studies in cancer cells have shown that p53 tumor suppressor protein is an important cellular stress sensor that triggers cell cycle stagnation and apoptosis, and also can modulate autophagy 65. In recent years, the sodium selenite, a selenide, has been speculated that it can regulate cancer cells by autophagy. Besides, some studies also found that selenide can induce autophagic cell death by promoting mitochondria damage 63. Liu et al confirmed that PTX can induce cell apoptosis in neuroblastoma via enhanced expression of LC3-II 66. Liu et al provided substantial evidence for autophagy induced by PTX 66. PTX can evidently increase LC3-II positive reaction point and the expression of autophagy-related proteins in endometrial cancer cells also increases significantly 67. Therefore, we hypothesized that selenized PTX may induce stronger autophagy than free PTX. Our study found that SePTX NPs could induce the expression of LC3-II on cancer cells. However, in normal cells, SePTX NPs did not significantly induce the expression of LC3-II, which suggests that the SePTX NPs-induced LC3-II protein expression has a specific effect on cancer cells. When autophagy occurs, LC3-I in the cytoplasm is recruited to the autophagosome to form LC3-II 68. Therefore, the LC3-II/LC3-I ratio is usually used as an indicator to reflect the level of autophagy in cells 68. In the present study, the western blot analysis found that ratio of LC3-II/LC3-I in Hela cells treated by SePTX NPs was up-regulated significantly. These results suggests that SePTX NPs can effectively activate the autophagy of cancer cells so as to promote the apoptosis of cancer cells, but have not obvious effects in normal cells.

*In vivo* experimental results showed that SePTX NPs had obvious anti-tumor effect, and its efficacy was similar to that of free PTX, which was consistent with MTT results. Interestingly, the body weight of mice in the control group increased gradually over time. About a week after the first injection, the body weight of mice in the free PTX group decreased significantly, suggesting that PTX has toxic effects *in vivo*. Compared with the control group, the body weight of mice in SePTX NPs group did not decrease significantly, but increased gradually. The change of body weight in SePTX NPs group was similar to that in the control group, which indicated that the *in vivo* toxicity of SePTX NPs was significantly less than that of free PTX. These results indicate that SePTX NPs not only have strong anti-tumor effect, but also have low *in vivo* toxicity.

## Conclusions

In summary, we revealed that SePTX NPs has no evident killing effect on normal cells. In cancer cells, SePTX NPs can successfully inhibit microtubule depolymerization, induce cell cycle arrest, which is related to up-regulation of microtubule associated protein p53 and cell cycle protein CyclinB1; on the other hand, SePTX NPs can successfully induce oxidative stress, cause mitochondrial dysfunction, induce mitochondrial pathway cell apoptosis, which is related to up-regulation of autophagy related protein LC3-II. In addition, SePTX NPs were able to significantly inhibit tumor growth in Lewis lung carcinoma tumor-bearing mice while reducing side effects of PTX. These results suggest that presently developed SePTX NPs have potential in the treatment of cancer.
